# Infectious Events Prior to Chemotherapy Initiation in Children with Acute Myeloid Leukemia

**DOI:** 10.1371/journal.pone.0061899

**Published:** 2013-04-26

**Authors:** Carol Portwine, David Mitchell, Donna Johnston, Biljana Gillmeister, Marie-Chantal Ethier, Rochelle Yanofsky, David Dix, Sonia Cellot, Victor Lewis, Victoria Price, Mariana Silva, Shayna Zelcer, Lynette Bowes, Bruno Michon, Kent Stobart, Josee Brossard, Joseph Beyene, Lillian Sung

**Affiliations:** 1 Hematology/Oncology, McMaster Children's Hospital at Hamilton Health Sciences, Hamilton, Ontario, Canada; 2 Hematology/Oncology, Montreal Children's Hospital, Montreal, Québec, Canada; 3 Hematology/Oncology, Children's Hospital of Eastern Ontario, Ottawa, Ontario, Canada; 4 Child Health Evaluative Sciences, The Hospital for Sick Children, Toronto, Ontario, Canada; 5 Hematology/Oncology, CancerCare Manitoba, Winnipeg, Manitoba, Canada; 6 Pediatric Hematology/Oncology, British Columbia Children's Hospital, Vancouver, British Columbia, Canada; 7 Hematology/Oncology, Hospital Sainte-Justine, Montreal, Quebec, Canada; 8 Hematology/Oncology/Transplant Program, Alberta Children's Hospital, Calgary, Alberta, Canada; 9 Pediatrics, IWK Health Centre, Halifax, Nova Scotia, Canada; 10 Hematology/Oncology, Cancer Centre of Southeastern Ontario at Kingston, Kingston, Ontario, Canada; 11 Hematology/Oncology, London Health Sciences, London, Ontario, Canada; 12 Hematology/Oncology, Janeway Child Health Centre, St. John's, Newfoundland, Canada; 13 Pediatric Hematology/Oncology Centre, Hospitalier Universitaire de Quebec Centre, Quebec City, Quebec, Canada; 14 Stollery Children's Hospital, University of Alberta Hospital, Edmonton Clinic Health Academy (ECHA), Edmonton, Alberta, Canada; 15 Hematology/Oncology, Centre Hospitalier Universitaire de Sherbrooke, Sherbrooke, Quebec, Canada; 16 Population Genomics Program, Department of Clinical Epidemiology and Biostatistics, McMaster University, Hamilton, Ontario, Canada; 17 Division of Haematology/Oncology, The Hospital for Sick Children, Toronto, Ontario, Canada; Health Protection Agency, United Kingdom

## Abstract

**Background:**

The primary objective was to describe infectious complications in children with acute myeloid leukemia from presentation to the healthcare system to initiation of chemotherapy and to describe how these infections differ depending on neutropenia.

**Methods:**

We conducted a retrospective, population-based cohort study that included children and adolescents with acute myeloid leukemia diagnosed and treated at 15 Canadian centers. We evaluated infections that occurred between presentation to the healthcare system (for symptoms that led to the diagnosis of acute myeloid leukemia) until initiation of chemotherapy.

**Results:**

Among 328 children, 92 (28.0%) were neutropenic at presentation. Eleven (3.4%) had sterile-site microbiologically documented infection and four had bacteremia (only one Gram negative). Infection rate was not influenced by neutropenia. No child died from an infectious cause prior to chemotherapy initiation.

**Conclusion:**

It may be reasonable to withhold empiric antibiotics in febrile non-neutropenic children with newly diagnosed acute myeloid leukemia until initiation of chemotherapy as long as they appear well without a clinical focus of infection. Future work could examine biomarkers or a clinical score to identify children presenting with leukemia and fever who are more likely to have an invasive infection.

## Introduction

Children with acute myeloid leukemia (AML) receive intensive chemotherapy and during treatment, they are at substantial risk of morbidity and mortality from invasive infections.[1] The management of fever with neutropenia is relatively straightforward in these children after they begin chemotherapy.[2] However, when children with acute leukemia initially enter the healthcare system, fever is a common presentation and neutropenia may also be present.[3] The optimal management of children with newly diagnosed leukemia with fever in the period preceding initiation of chemotherapy is uncertain, both for those with and without neutropenia. Cancer-specific fever and neutropenia guidelines do not provide recommendations for this scenario since the setting for guidelines is usually restricted to patients who are receiving treatments for cancer.[2], [4] Furthermore, it would be useful to know if infection outcomes differed depending on the presence or absence of neutropenia in this setting since the presence of neutropenia is a major criterion that influences the decision to start antibiotics in febrile children receiving treatment for cancer.

Consequently, we conducted a population-based study of pediatric AML using a retrospective cohort design. The primary objective was to describe infectious complications in children with AML from presentation to the healthcare system to initiation of chemotherapy and to describe how these infections differ depending on neutropenia.

## Materials and Methods

### Ethics Statement

This study was approved by the Research Ethics Board at The Hospital for Sick Children and local Research Ethics Boards of the 14 other participating sites (McMaster University-Hamilton Health Sciences/Faculty of Health Sciences Research Ethics Board, Montreal Children's Hospital Research Ethics Board, Children's Hospital of Eastern Ontario Research Ethics Board, University of Winnipeg Research Ethics Board, University of British Columbia/Children's and Women's Health Centre of British Columbia Research Ethics Board, Centre Hospitalier Universitaire Sainte-Justine Research Ethics Board, University of Calgary Conjoint Health Research Ethics Board, IWK Research Ethics Board, Queen's University-Health Sciences Research Ethics Board, University of Western Ontario Research Ethics Board for Health Science Research Involving Human Subjects, Memorial University Human Investigation Committee, Centre Hospitalier Universitaire de Quebec Research Ethics Board, University of Alberta Health Research Ethics Board-Biomedical Panel, and Centre Hospitalier Universitaire de Sherbrooke Research Ethics Board). As this was a retrospective review study, the Research Ethics Board at The Hospital for Sick Children and those at the 14 other participating sites waived the need for written informed consent. All demographic information and information about the child's diagnosis and treatment were abstracted from the child's chart.

### Study Sample

This manuscript is related to a large retrospective, population-based cohort study in which we included children and adolescents with AML diagnosed and treated in each Canadian province except Saskatchewan.[5] We included children and adolescents ≤18 years with *de novo* AML diagnosed between January 1, 1995 and December 31, 2004. Children with Down syndrome were also included. We excluded those with acute promyelocytic leukemia, secondary AML, and previous diagnosis of immunodeficiency.

### Outcome Measures

We evaluated infections that occurred between presentation to the healthcare system (for symptoms that led to the diagnosis of AML) until initiation of chemotherapy. Data abstraction was conducted by trained clinical research associates who travelled to each site to abstract and code all data.

We described the occurrence of sterile site invasive infection[6], clinically documented infection and fever of unknown origin. Fever was defined as a single oral temperature of 38.3° C or a temperature of 38.0° C for 1 hour.[7] In this study, we did not distinguish between infections present at the time of admission and those acquired after admission but prior to initiation of treatment. Positive cultures with common contaminants such as coagulase negative *Staphylococcus* were only considered true infection if there were two or more positive cultures in the same episode or if the infection was associated with sepsis. In this study, sepsis was defined as systemic inflammatory response syndrome in the presence of suspected or proven infection and organ dysfunction.[8] Clinically documented infections were classified based upon the Centers for Disease Control and Prevention (CDC) definitions of nosocomial infections.[9] However, for our study, we did not require that clinically documented infections be not present or incubating at the time of admission to hospital. Fever of unknown origin was fever occurring in the absence of a positive microbiology result or clinical site of infection.

### Potential Predictors

We compared baseline characteristics and infection outcomes depending upon if the patient was neutropenic (absolute neutrophil count (ANC)<0.5×10^9^/L) at presentation.

Characteristics evaluated were: gender, age, Down syndrome, body mass index percentile at diagnosis, peripheral blood counts at diagnosis and AML morphology. Obesity was defined as body mass index (BMI) ≥95th percentile and underweight was defined as BMI ≤10th percentile for age and gender according to the CDC for those at least 2 years of age.[10] BMI was evaluated as obesity previously has been associated with treatment-related mortality and infections in pediatric AML[11], [12].

### Statistics

Characteristics and infection outcomes were compared between those who were and were not neutropenic at presentation using the Wilcoxon rank sum test for continuous variables and Chi square or Fisher's exact test for categorical variables. All tests of significance were two-sided, and statistical significance was defined as P<0.05. Statistical analysis was performed using the SAS statistical program (SAS-PC, version 9·3; SAS Institute Inc., Cary, NC).

## Results

There were 343 children with de novo AML who met eligibility criteria in the study time frame. Of these children, 15 began chemotherapy on the same day as presentation to the healthcare center and thus, there were 328 children with pre-chemotherapy information available. The median time between presentation and initiation of chemotherapy was 2 (range 0 to 274) days. There were two patients who had very lengthy delays between presentation and initiation of chemotherapy of 59 and 274 days.

Table S1 illustrates that 92 (28.0%) children were neutropenic at presentation. Gender and age were not significantly associated with neutropenia at presentation whereas children with Down syndrome were significantly less likely to present with neutropenia. Children with neutropenia had a significantly lower initial peripheral blast count and hemoglobin level compared to non-neutropenic children although the initial platelet count was similar between the two groups.

Table 1 and Table S2 illustrate the infection outcomes. Overall, there were 11/328 (3.4%) children who experienced 12 sterile site microbiologically documented infections; the risk did not significantly differ by the presence or absence of neutropenia. There were four episodes of bacteremia with viridans group streptococci (n = 3) and *Pseudomonas aeruginosa* (n = 1), all occurring in non-neutropenic children. Among the six episodes of urinary tract infection, the causative agents were: *Enterococcus* species (n = 2), *Escherichia coli* (n = 2), *P. aeruginosa* (n = 1) and *Candida albicans* (n = 1). The two other sterile site infections were *Staphylococcus aureus* from a peritoneal drain (see below) and a lymph node biopsy. Other than for the one *C. albicans* urinary tract infection, no other fungi from sterile or non-sterile sites were observed (data not shown). One child experienced sepsis with concurrent pneumonia.

**Table 1:** Infection outcomes before chemotherapy initiation by neutropenia at presentation.*

	Neutropenic at Presentation (N = 92)	Not Neutropenic at Presentation (N = 236)	P Value
Median days of fever (range)	1.0 (0.0–17.0)	1.0 (0.0–16.0)	0.794
Sterile site microbiologically documented infection (%)	2 (2.2)	9 (3.8)	0.734
Gram-positive sterile site infection (%)	1 (1.1)	6 (2.5)	0.678
Gram-negative sterile site infection (%)	1 (1.1)	3 (1.3)	1.000
Bacteremia (%)^a^	0	4 (1.7)	0.580
Invasive fungal infection (%)	1 (2.2)	0	0.281
Clinically documented infection (%)	12 (13.0)	31 (13.1)	1.000
Fever of unknown origin (%)	35 (38.0)	86 (36.4)	0.886
Median days intravenous antibiotics (range)	1.0 (0.0–16.0)	0.0 (0.0–25.0)	0.073
Sepsis (%)	1.0 (1.1)	0	0.281
Infection-related mortality (%)	0	0	NC

Abbreviation: NC–not calculable.

* Infection outcomes denote at least one occurrence during the pre-chemotherapy period.

a Bacteremias were viridans group streptococci (n = 3) and *Pseudomona aeruginosa* (n = 1).

Three children died before initiation of chemotherapy. Two children died of hyperleukocytosis and pulmonary leukostasis. A third child presented with a 10 day history of fever and was found to have pneumonia on chest radiograph; the absolute neutrophil count was 0.2×10^9^/L at presentation. The child was treated with broad-spectrum antibiotics and after 10 days of persistent fever, she was found to have *C. albicans* from urine and therefore, amphotericin B was initiated. Over the next four days, she developed acute respiratory distress and multi-organ failure; a peritoneal drain grew *S. aureus*. Autopsy failed to reveal an infectious cause of death and death was attributed to progressive AML.

## Discussion

We conducted a comprehensive assessment of infections occurring before chemotherapy initiation in newly diagnosed pediatric AML. We found that sterile site infections were rare, Gram negative bacteremia occurred in only one child, and many infections were from a urinary site. Infection outcomes were similar between neutropenic and non-neutropenic children. Our findings suggest that it may be reasonable to withhold empiric antibiotics in febrile non-neutropenic children with newly diagnosed AML until initiation of chemotherapy as long as they appear well without a clinical focus of infection. Reducing antibiotic exposure is important given the relationship between greater antibiotic use and antibiotic resistance[13], [14] and invasive fungal infection.[15] However, the safety of this approach has not yet been tested.

Although our analysis did not show a difference in outcomes by neutropenia at presentation, limited power for this comparison is an important consideration since only 28% of children were neutropenic. Patients with non-cancer related neutropenia may be at risk of invasive infection if the neutropenia is associated with profound immunosuppression.[16] Thus, we do not suggest withholding empiric antibiotics in this situation and suggest empiric antibiotics be initiated in a febrile neutropenic child with newly diagnosed AML. Our data also suggest that if empiric antibiotics are initiated (for example, because of neutropenia, unwell appearance or a clinical site of infection), broad-spectrum antibiotics with activity against *P.aeruginosa* are important.

Future work could examine biomarkers or a clinical score to identify children presenting with leukemia and fever who are more likely to have an invasive infection. This approach may allow antibiotic therapy to be tailored to children at higher risk of infection.

The strengths of our report are the multi-center nature of the study which allowed capture of a large number of children with AML. Further, the data are highly generalizable. However, there are important limitations to our study. Centers had different approaches to supportive care and urgency of chemotherapy initiation. Second, the retrospective design made it more difficult to appreciate the clinical status of the child at presentation.

In conclusion, we found that in children with AML, 28% will present with neutropenia. Sterile site infections were rare. It may be reasonable to withhold empiric antibiotics in febrile non-neutropenic children with newly diagnosed AML until initiation of chemotherapy as long as they appear well without a clinical focus of infection.

## Supporting Information

Table S1 Characteristics of children with acute myeloid leukemia with pre-chemotherapy information available by neutropenia at presentation.
(DOC)

Table S2 Microbiologically documented sterile site infections observed between presentation to the healthcare center until chemotherapy initiation.
(DOC)
