# Supplementary material for: Infectious Events Prior to Chemotherapy Initiation in Children with Acute Myeloid Leukemia
Source: PLoS One. 2013 Apr 26;8(4):e61899. doi: 10.1371/journal.pone.0061899 (PMC3637321; doi:10.1371/journal.pone.0061899)
Supplement: Table S1 — Characteristics of children with acute myeloid leukemia with pre-chemotherapy information available by neutropenia at presentation. (DOC) [file pone.0061899.s001.doc]

Table S1: Characteristics of children with acute myeloid leukemia with pre-chemotherapy information available by neutropenia at presentation

|  | **Neutropenic at Presentation**  **(N=92)** | **Not Neutropenic at Presentation (N=236)** | **P Value** |
| --- | --- | --- | --- |
| Male (%) | 39 (42.4) | 124 (52.5) | 0.126 |
| Median age in years (range) | 8.6 (0.3-18.0) | 6.8 (0.04-17.3) | 0.081 |
| Down syndrome (%) | 3 (3.3) | 27 (11.4) | 0.036 |
| Body mass index (%)^a^ |  |  | 0.209 |
| Obese | 6 (8.2) | 23 (13.7) |  |
| Normal weight | 59 (80.1) | 133 (79.2) |  |
| Underweight | 8 (11.0) | 12 (7.1) |  |
| Median white blood cell count at diagnosis (x10^9^/L) (range) | 5.6 (0.4-421.3) | 22.1 (2.2-496.0) | <0.0001 |
| Median peripheral blast at diagnosis (x10^9^/L) (range) | 0.7 (0.0-412.9) | 4.3 (0.0-689.9) | 0.0003 |
| Median hemoglobin at diagnosis (gm/L) (range) | 78.0 (31.0-135.0) | 85.0 (24.0-171.0) | 0.047 |
| Median platelet count at diagnosis (x10^9^/L) (range) | 49.0 (3.0-361.0) | 53.0 (0.0-549.0) | 0.634 |
| FAB AML Morphology (%) |  |  | <0.0001 |
| M0 | 4 (4.3) | 2 (0.8) |  |
| M1 | 18 (19.6) | 30 (12.7) |  |
| M2 | 14 (15.2) | 64 (27.1) |  |
| M4 | 17 (18.5) | 47 (19.9) |  |
| M5 | 29 (31.5) | 30 (12.7) |  |
| M6 | 1 (1.1) | 6 (2.5) |  |
| M7 | 2 (2.2) | 38 (16.1) |  |
| Other^b^ | 7 (7.6) | 19 (8.1) |  |

Abbreviation: AML – acute myeloid leukemia

^a^ Only available for children ≥ 2 years of age

^b^ Other includes: AML with multilineage dysplasia (n=1), isolated granulocytic sarcoma (chloroma) (n=1) and AML not further classified (n=5) for neutropenic group; and isolated granulocytic sarcoma (chloroma) (n=1), AML biphenotypic (n=2), AML with multilineage dysplasia (n=2) and AML not further classified (n=14) for the not neutropenic group
