# Supplementary material for: Infectious Events Prior to Chemotherapy Initiation in Children with Acute Myeloid Leukemia
Source: PLoS One. 2013 Apr 26;8(4):e61899. doi: 10.1371/journal.pone.0061899 (PMC3637321; doi:10.1371/journal.pone.0061899)
Supplement: Table S2 — Microbiologically documented sterile site infections observed between presentation to the healthcare center until chemotherapy initiation. (DOC) [file pone.0061899.s002.doc]

**Table S2: Microbiologically documented sterile site infections observed between presentation to the healthcare center until chemotherapy initiation***

|  | **Neutropenic at Presentation**  **(n=3)** | **Not Neutropenic at Presentation (n=9)** |
| --- | --- | --- |
| Bacteria |  |  |
| Gram-positive |  |  |
| Viridans group streptococci | 0 | 3 |
| *Enterococcus* species | 0 | 2 |
| *Staphylococcus aureus* | 1 | 1 |
|  |  |  |
| Gram-negative |  |  |
| *Escherichia coli* | 1 | 1 |
| *Pseudomonas aeruginosa* | 0 | 2 |
|  |  |  |
| Fungus |  |  |
| *Candida albicans* | 1 | 0 |

* Sites were blood: viridans group streptococci (n=3) *and Pseudomonas aeruginosa* (n=1); urine: *Enterococcus* species (n=2), *Escherichia coli* (n=2), *Pseudomonas aeruginosa* (n=1), and *Candida albicans* (n=1); peritoneal fluid: *Staphylococcus aureus* (n=1); and lymph node biopsy: *S. aureus* (n=1)
